# Exploring Determinants of Mediterranean Lifestyle Adherence: Findings from the Multinational MEDIET4ALL e-Survey Across Ten Mediterranean and Neighboring Countries

**DOI:** 10.3390/nu17142280

**Published:** 2025-07-10

**Authors:** Achraf Ammar, Mohamed Ali Boujelbane, Atef Salem, Khaled Trabelsi, Bassem Bouaziz, Mohamed Kerkeni, Liwa Masmoudi, Juliane Heydenreich, Christiana Schallhorn, Gabriel Müller, Ayse Merve Uyar, Hadeel Ali Ghazzawi, Adam Tawfiq Amawi, Bekir Erhan Orhan, Giuseppe Grosso, Osama Abdelkarim, Mohamed Aly, Tarak Driss, Kais El Abed, Wassim Moalla, Piotr Zmijewski, Frédéric Debeaufort, Nasreddine Benbettaieb, Clément Poulain, Laura Reyes, Amparo Gamero, Marta Cuenca-Ortolá, Antonio Cilla, Nicola Francesca, Concetta Maria Messina, Enrico Viola, Björn Lorenzen, Stefania Filice, Aadil Bajoub, El-Mehdi Ajal, El Amine Ajal, Majdouline Obtel, Sadjia Lahiani, Taha Khaldi, Nafaa Souissi, Omar Boukhris, Waqar Husain, Evelyn Frias-Toral, Walid Mahdi, Hamdi Chtourou, Haitham Jahrami, Wolfgang I. Schöllhorn

**Affiliations:** 1 Department of Training and Movement Science, Institute of Sport Science, Johannes Gutenberg-University Mainz, 55122 Mainz, Germany; mboujelb@uni-mainz.de (M.A.B.); asalem@uni-mainz.de (A.S.); gabriel.mueller@uni-mainz.de (G.M.); auyar@students.uni-mainz.de (A.M.U.); schoellw@uni-mainz.de (W.I.S.); 2 Research Laboratory, Molecular Bases of Human Pathology, LR19ES13, Faculty of Medicine of Sfax, University of Sfax, Sfax 3000, Tunisia; nafaa.souissi@isseps.usf.tn; 3 High Institute of Sport and Physical Education of Sfax, University of Sfax, Sfax 3000, Tunisia; mohamedkerkeni24@yahoo.com (M.K.); liwa.masmoudi@isseps.usf.tn (L.M.); kais.elabed@gmail.com (K.E.A.); wassim.moalla@isseps.usf.tn (W.M.); h_chtourou@yahoo.fr (H.C.); 4 Department of Movement Sciences and Sports Training, School of Sport Science, The University of Jordan, Amman 11942, Jordan; trabelsikhaled@gmail.com (K.T.); a.amawi@ju.edu.jo (A.T.A.); 5 Research Laboratory: Education, Motricity, Sport and Health, EM2S, LR19JS01, High Institute of Sport and Physical Education of Sfax, University of Sfax, Sfax 3000, Tunisia; 6 Multimedia Information Systems and Advanced Computing Laboratory (MIRACL), University of Sfax, Sfax 3021, Tunisia; bassem.bouaziz@isims.usf.tn (B.B.); walid.mahdi@isims.usf.tn (W.M.); 7 Higher Institute of Information Science and Multimedia of Sfax (ISIMS), University of Sfax, Sfax 3021, Tunisia; 8 Department of Experimental Sports Nutrition, Faculty of Sports Sciences, Leipzig University, 04109 Leipzig, Germany; juliane.heydenreich@uni-leipzig.de; 9 Department of Sports Economics, Sociology and History, Institute of Sport Science, Johannes Gutenberg-University Mainz, 55122 Mainz, Germany; christiana.schallhorn@uni-mainz.de; 10 Department of Nutrition and Food Technology, School of Agriculture, The University of Jordan, Amman 11942, Jordan; h.ghazzawi@ju.edu.jo; 11 Faculty of Sports Sciences, Istanbul Aydın University, 34295 Istanbul, Turkey; bekirerhanorhan@aydin.edu.tr; 12 Department of Biomedical and Biotechnological Sciences, University of Catania, 95123 Catania, Italy; giuseppegrosso82@gmail.com; 13 Faculty of Sport Sciences, Assiut University, Assiut 71515, Egypt; osamahalim@ymail.com (O.A.); mohamed.aly@aun.edu.eg (M.A.); 14 ESLSCA University Egypt, Giza 12676, Egypt; 15 Interdisciplinary Laboratory in Neurosciences, Physiology, and Psychology: Physical Activity, Health, and Learning (LINP2), UFR STAPS, Paris Nanterre University, 92000 Nanterre, France; tarak.driss@parisnanterre.fr; 16 Department of Biomedical Sciences, Jozef Pilsudski University of Physical Education in Warsaw, 00-968 Warsaw, Poland; piotr.zmijewski@insp.waw.pl; 17 Department BioEngineering, Institut Universitaire de Technologie IUT-Dijon, Université Bougogne Europe, Blvd Dr. Petitjean, CEDEX, 21078 Dijon, France; frederic.debeaufort@u-bourgogne.fr (F.D.); nasreddine.benbettaieb@u-bourgogne.fr (N.B.); clement.poulain@u-bourgogne.fr (C.P.); 18 Joint Research Unit UMR PAM-PCAV (Physical-Chemistry of Food and Wine Laboratory), Université Bourgogne Europe/Institut AgroDijon/INRAE, 1 Esplanade Erasme, 21000 Dijon, France; 19 Vitagora Innovation Cluster, 21000 Dijon, France; laura.reyes@vitagora.com; 20 Department of Preventive Medicine and Public Health, Food Science, Toxicology and Forensic Medicine, Faculty of Pharmacy & Food Sciences, University of Valencia, 46100 Valencia, Spain; amparo.gamero@uv.es (A.G.); marta.cuenca@uv.es (M.C.-O.); antonio.cilla@uv.es (A.C.); 21 Department of Agricultural Food and Forest Sciences, University of Palermo, Viale delle Scienze, 90128 Palermo, Italy; nicola.francesca@unipa.it (N.F.); enrico.viola01@unipa.it (E.V.); 22 Laboratory of Marine Biochemistry and Ecotoxicology, Department of Earth and Marine Sciences DiSTeM, University of Palermo, Via Barlotta 4, 91100 Trapani, Italy; concetta.messina@unipa.it; 23 Microtarians Academy, 1746 Luxembourg, Luxembourg; b.lorenzen@microtarians.com (B.L.); stefania.filice@microtarians.com (S.F.); 24 Laboratory of Food and Food By-Products Chemistry and Processing Technology, National School of Agriculture in Meknès, km 10, Haj Kaddour Road, B.P. S/40, Meknès 50001, Morocco; aliam80@hotmail.com (A.B.); elmehdi_ajal@um5.ac.ma (E.-M.A.); 25 Laboratory of Social Medicine, Department of Epidemiology and Public Health, Faculty of Medicine and Pharmacy of Rabat, Mohammed V University, Rabat 10100, Morocco; m.obtel@um5r.ac.ma; 26 UPR of Pharmacognosy, Faculty of Medicine and Pharmacy of Rabat, Mohammed V University, Rabat 10100, Morocco; amine_266@hotmail.com; 27 VALCORE Laboratory, Department of Biology, Faculty of Science, University of M’hamed Bougara Boumerdes, Boumerdes 35000, Algeria; sadjialahiani@yahoo.fr; 28 Biotechnology Research Center CRBt, Constantine 25000, Algeria; t.khaldi@crbt.dz; 29 SIESTA Research Group, School of Allied Health, Human Services and Sport, La Trobe University, Melbourne 3086, Australia; o.boukhris@latrobe.edu.au; 30 Sport, Performance, and Nutrition Research Group, School of Allied Health, Human Services and Sport, La Trobe University, Melbourne 3086, Australia; 31 Department of Humanities, COMSATS University Islamabad, Islamabad Campus, Park Road, Islamabad 45550, Pakistan; drsukoon@gmail.com; 32 Escuela de Medicina, Universidad Espíritu Santo, Samborondón 0901952, Ecuador; 33 Division of Research, Texas State University, 601 University Dr, San Marcos, TX 78666, USA; 34 Research Unit, Physical Activity, Sport, and Health, UR18JS01, National Observatory of Sport, Tunis 1003, Tunisia; 35 Government Hospitals, Manama P.O. Box 12, Bahrain; 36 Department of Psychiatry, College of Medicine and Health Sciences, Arabian Gulf University, Manama P.O. Box 26671, Bahrain

**Keywords:** Mediterranean diet, public health, food literacy, nutrition knowledge, exercise, active lifestyle, socialization, engagement, food, multicenter study

## Abstract

**Background/Objectives:** Despite its well-established health benefits, adherence to the Mediterranean lifestyle (MedLife) has declined globally, including in its region of origin, alongside a significant shift toward ultra-processed food consumption. Understanding the factors associated with MedLife adherence is essential for developing targeted interventions and tailored policy recommendations. As part of the MEDIET4ALL PRIMA project, this cross-sectional study aimed to comprehensively examine geo-demographic, socio-economic, psychological, behavioral, and barrier-related factors associated with and potentially contributing to MedLife adherence. **Methods:** Data were collected from 4010 participants aged 18 years and above across ten Mediterranean and neighboring countries using the multinational MEDIET4ALL e-survey, which included the validated MedLife index, along with various other questionnaires. **Results:** Results indicate that only 22% of respondents demonstrated high adherence to the Mediterranean lifestyle (MedLife), with significant variability observed across countries, age groups, education levels, and health statuses. Spain had the highest proportion of participants with high adherence (38%). Factors associated with significantly higher adherence rates include older age, living in the Mediterranean region, higher education levels, a greater awareness of MedLife principles, lower perceived barriers, normal BMI, better health status, and stable economic and marital conditions (*p*-values ranging from 0.04 to <0.001). Additionally, individuals with high MedLife adherence exhibited more socially and physically active lifestyles and experienced less psychological strain (*p* < 0.001). Regression analyses identified MedLife awareness as the strongest positive predictor of adherence (β = 0.206), followed by social participation (β = 0.194) and physical activity (β = 0.096). Additional positive contributors include life satisfaction, sleep quality, living in the Mediterranean region, age, and education (β ranging from 0.049 to 0.093). Conversely, factors that are negatively associated with adherence include sedentary behavior, living environment, and barriers such as low motivation, taste dislike, price unaffordability, limited availability, and the time-consuming nature of preparing Mediterranean food (MedFood; β ranging from −0.036 to −0.067). **Conclusions:** These findings indicate that fewer than one in four adults across Mediterranean and neighboring countries demonstrate high adherence to MedLife, supporting prior evidence of suboptimal adherence even within Mediterranean regions. This study identified a range of behavioral, socio-demographic, and environmental factors—both positive and negative predictors—that can help guide the design of targeted, culturally adapted interventions to promote MedLife behavior. Future research should incorporate objective measurements and longitudinal monitoring to better understand underlying mechanisms, establish causality, and develop sustainable strategies for enhancing MedLife adherence in diverse populations.

## 1. Introduction

The Mediterranean lifestyle (MedLife) is a holistic model that blends diet, daily activity, and the social–cultural practices typical of Mediterranean countries [1,2]. Its dietary core—the Mediterranean diet (MedDiet)—centers on fresh, minimally processed foods: plentiful fruits, vegetables, whole grains, legumes, nuts, and olive oil; moderate fish and dairy; and minimal red meat or processed items [3]. Modern MedDiet pyramids also highlight regular exercise, shared meals, and other psychosocial traditions, underscoring how diet, movement, and community together support well-being and longevity [1,2]. Indeed, MedLife is considered the most evidence-backed eating and lifestyle pattern, benefiting both health and the planet by fostering biodiversity, conserving water and energy, cutting emissions, and supporting local economies while honoring cultural values [4,5]. Experts also rank it among the easiest healthy patterns to adopt because of its food diversity and socio-cultural appeal [2,4]. Adherence brings numerous benefits [6]: lower risks of cardiovascular disease, obesity, diabetes, and several cancers [7]; better mental health and longer life expectancy [8,9]; and roughly a 25% drop in all-cause mortality [10]. UNESCO’s designation of the Mediterranean diet as intangible cultural heritage further underscores its health, cultural, and ecological importance [11].

However, despite these well-documented benefits, adherence to MedLife has been on the decline in recent decades, both globally and within Mediterranean regions where the diet originated [12,13]. A significant driver of this decline is the rising consumption of ultra-processed foods (UPFDs) [14]. Compared to unprocessed or minimally processed foods such as the MedDiet, UPFDs are nutritionally imbalanced, energy-dense, and associated with higher risks of obesity, cardiovascular diseases, and overall mortality [14,15,16]. The exponential growth in the availability and consumption of UPFDs and takeout food in recent decades has further exacerbated the issue, even in Mediterranean regions [17]. In several EUR-MED countries, highly processed foods contribute to over 50% of nutrient intake, with particularly high levels reported in Spain (61%) and Germany (79%) [18]. This shift away from traditional dietary patterns toward industrialized options is largely driven by modern societal trends, including the convenience and profitability-driven global industrial food system. These food choices are often unhealthy, highly accessible, and aggressively marketed [14]. Additionally, factors such as the proliferation of Western dietary patterns [19], the reduced availability of traditional MedDiet ingredients, high costs [20], a lack of modernization in food preparation methods, and low awareness of the MedDiet’s health benefits [1] could further contribute to this alarming trend.

This growing divergence from MedLife emphasizes the urgent need to investigate the determinants of adherence to MedLife. Adherence to MedLife appears to be influenced by a complex interplay of demographic, socio-economic, psychological, geographic, behavioral, and cultural factors, many of which remain poorly understood [12]. Existing research has identified several key determinants influencing adherence [21]. For example, socio-demographic factors such as age, gender, income, and education level play significant roles. Individuals with higher education and income levels tend to adhere more closely to MedLife principles, as these factors are strongly associated with greater awareness and access to healthy dietary patterns such as the MedDiet [22]. In the same context, Bonaccio et al. [23] and Papadaki et al. [24] argue that socio-economic factors significantly contribute to the shift from the MedDiet toward more Westernized diets and an increased consumption of convenience foods, with their studies showing a positive relationship between MedDiet adherence and socio-economic status. Nevertheless, the impact of demographic characteristics such as age and sex remains unclear. Some studies have reported a decline in adherence with age [25,26,27] due to factors such as a loss of interest in food, chewing difficulties, financial hardships after retirement, prevalence of cognitive decline, and/or dependency in food preparation. Conversely, others have observed an increase in adherence with age, possibly driven by the prevalence of nutrition-related disorders requiring dietary modifications that align with MedDiet principles [28]. Similarly, findings on sex differences in adherence to the MedDiet have been inconsistent. While some studies report better adherence among women—primarily attributed to lower red meat consumption [29]—others have found no significant differences between men and women [30]. Psychological factors, such as motivation and perceived barriers, also play a critical role in shaping adherence. For example, individuals with higher health motivation are more likely to adopt MedLife practices [7]. Furthermore, lifestyle behaviors, including regular physical activity, social engagement, and adequate sleep, have been shown to positively influence adherence [8,21].

Despite these insights, uncertainty still surrounds the direction and strength of the association of certain factors, such as age and sex, with MedLife adherence, as the literature offers inconsistent findings that may stem from methodological variations, cultural differences, or a lack of integrated analysis of relevant covariates. More importantly, substantial gaps persist in our understanding of how socio-demographic, psychological, and behavioral variables interact and jointly contribute to adherence across diverse populations and cultural contexts [19]. Prior research has tended to examine these determinants in isolation or within specific demographic groups, limiting the generalizability and contextual relevance of findings [21,22,23,24,25,26,27,28,29,30]. Furthermore, in light of the rapid global evolution of dietary patterns and lifestyle behaviors, particularly in Euro-Mediterranean countries, there is a pressing need for a comprehensive and up-to-date assessment of the multifactorial determinants influencing adherence to a healthy lifestyle. Addressing this gap requires an integrative approach that considers geographic, psychological, behavioral, and environmental dimensions to better understand how these factors interact to shape adherence to MedLife principles.

This study, conducted as part of the “MEDIET4ALL” PRIMA project entitled “Transnational Movement to Support the Sustainable Transition towards a Healthy and Eco-friendly Agri-Food System through the Promotion of MEDIET and its Lifestyle in Modern Society,” and supported by the European Union [31], seeks to address these gaps. It provides a comprehensive analysis of geo-demographic and socio-economic characteristics, psychological variables, perceived barriers, and lifestyle behaviors associated with and potentially contributing to MedLife adherence across different adult age groups in ten Mediterranean and neighboring countries.

By targeting a large sample size and employing a comprehensive set of validated sociodemographic, environmental, psychosocial, and behavioral questionnaires—including validated MedDiet adherence scores, which align with the principles of the MEDIET Pyramid and are applicable to the general population [32,33]—this research aims to identify significant predictors of adherence and explore the interplay between these factors in shaping adherence patterns [8]. Such insights would support the development of future tailored, evidence-based strategies to promote adherence to MedLife, while aligning with contemporary socio-cultural and behavioral trends in the Euro-Mediterranean region [34].

## 2. Materials and Methods

### 2.1. Survey Development and Participant Recruitment

This cross-sectional study assessed adherence to MedDiet and MedLife, along with potential determinant factors, using the MEDIET4ALL international electronic survey. The survey was designed, reviewed, and edited by a multidisciplinary team of researchers—including experts in public health, nutrition, sports and movement sciences, psychology, and sociology—at Johannes Gutenberg University of Mainz, Germany, in collaboration with partner universities involved in the MEDIET4ALL PRIMA project, supported by the European Union [35,36].

Prior to its dissemination, the survey underwent a one-week pilot testing phase conducted by the project’s steering group. Following revisions based on feedback, the finalized survey was distributed during the summer of 2024 for a period of four months across seven Mediterranean countries (Spain, Italy, France, Turkey, Tunisia, Algeria, and Morocco) and three non-Mediterranean countries (Germany, Luxembourg, and Jordan), grouped based on strict geographic and historical definitions of the Mediterranean [25,35,36]. The main adjustments involved linguistic refinements aimed at improving clarity and comprehension across the different language versions—particularly for those questionnaires that did not yet have fully validated translations. These revisions ensured consistency in terminology and question phrasing, especially for culturally sensitive items and complex behavioral questions. As the instruments were primarily based on standardized and validated items, no structural changes were made to the core content.

Various organizations from Europe, North Africa, and Western Asia facilitated the survey’s promotion and administration [35,36].

To ensure accessibility, the survey was available in seven languages: English, German, French, Italian, Spanish, Arabic, and Turkish. Items without official translations were rigorously translated and back-translated, ensuring excellent test–retest reliability coefficients (r = 0.81–0.94) for all translated items [35,36]. The survey contained 75 questions derived from validated questionnaires, assessing MedLife adherence, potential barriers, and contributing factors such as geo-demographic and socio-economic characteristics, health status, mental health, life satisfaction, and multidimensional lifestyle behaviors (e.g., physical activity, social participation, sleep, and technology use) [35,36]. Completion of the survey was estimated to take 15–20 min.

The survey was hosted on the SoSci Survey platform, (https://www.soscisurvey.de/en/index) a “General Data Protection Regulation” (GDPR) compliant web application, supported by the services of Johannes Gutenberg University. A link to the survey was disseminated by the MEDIET4ALL consortium and collaborators (e.g., Bilendi solution) through various channels, including email invitations, official university and consortium web pages, the MEDIET4ALL website, and social media platforms such as ResearchGate™, LinkedIn™, Facebook™, WhatsApp™, and Twitter™. Additionally, members of the general public were encouraged to promote the survey within their personal networks.

The survey began with an introductory page outlining the study’s background, objectives, ethics, data privacy, and consent information, as well as the option to select one of the seven available languages. The survey initially attracted over 8000 participants from diverse regions. After rigorous screening for validity and completeness, 4010 responses were included in the final analysis. Completeness was assessed based on the overall response rate, with only fully completed surveys being retained, while responses with missing data were excluded. Validity checks and logic-based screening were conducted to identify inconsistent or contradictory responses, such as indicating “no vigorous physical activity” while simultaneously reporting daily vigorous exercise. Additionally, duplicate responses were identified and excluded based on a combination of factors, including identical IP addresses, near-identical timestamps, and highly similar demographic and survey responses. Responses containing extreme or unrealistic values, such as reporting excessive sleep durations (e.g., 24 h) or implausible food intake patterns, were also removed to ensure data accuracy and reliability. A broad, general population sample was targeted to ensure diversity and enhance the statistical power of analyses.

The study was conducted in compliance with the principles outlined in the Declaration of Helsinki. The protocol and consent form were approved by the Ethics Committee of the Faculty of Medicine at the University of Sfax (approval identification code: 058/24).

### 2.2. Data Privacy and Consent of Participation

Participation in this study was entirely voluntary, with no justification required for declining to participate, withdrawing, or ceasing participation at any stage. To ensure ethical compliance, participants were informed that (i) all collected data would be used solely for research purposes and (ii) responses would remain anonymous and confidential in accordance with the SoSci Survey privacy policy (www.soscisurvey.de/en/privacy (accessed on 1 May 2024)). The survey was hosted on the services of Johannes Gutenberg University and adhered to the Federal Data Protection Act (BDSG) and the EU General Data Protection Regulation (GDPR).

Participants were not asked to provide any personally identifiable information, such as names or contact details. Furthermore, participants had the option to withdraw from the survey at any point before submission, and in such cases, their responses were not saved. Responses were recorded only when participants actively submitted the questionnaire by clicking the “submit” button.

The study ensured that opting out or withdrawing consent at any stage did not entail any negative consequences for participants. By completing and submitting the questionnaire, participants provided informed consent for the anonymous use of their data for research purposes.

### 2.3. Survey Questionnaires

The survey incorporated a variety of validated questionnaires as well as other specific questions to comprehensively assess adherence to the MedLifestyle, alongside associated factors.

#### 2.3.1. MedLife Index

Available MedDiet adherence scores were recently evaluated in a systematic review by Zaragoza-Martí et al. [37]. According to this review, 12 of the 28 scores analyzed were applied to the general population, 5 followed the principles of the MEDIET Pyramid [1,2], but only the score “MEDLIFE index” created by Sotos-Prieto et al. [33] provided a good internal consistency by Cronbach’s α coefficient of 0.75. Accordingly, the MEDLIFE index has been elected to be part of the MEDIET4ALL e-survey. This questionnaire is a validated tool designed to evaluate adherence to MedDiet and MedLife habits in the general population and is based on the principles of the MedDiet pyramid. It includes 28 items grouped into three categories: (i) Food consumption frequency (15 items), which evaluates adherence to MedDiet patterns, such as a limited intake of pastries and red meat, and the adequate consumption of fruits, vegetables, whole grains, and healthy fats, (ii) MedDiet habits (7 items), which assess behaviors like limiting salt, sugar, and snacking between meals, and (iii) lifestyle behaviors (6 items), which assess physical activity (e.g., ≥150 min of moderate activity per week), rest (6–8 h of sleep), social habits, and conviviality.

Each item is scored as 0 (non-adherence) or 1 (adherence), with a total score ranging from 0 (worst adherence) to 28 (best adherence). Scores are categorized into three levels for interpretation: low (<12), medium (12–16), and high (>16). The score’s thresholds for categorizing adherence levels (low, medium, and high) are based on the tertiles (or quantiles) of the total scores in the entire dataset. This index provides a comprehensive measure of dietary and lifestyle adherence to the Mediterranean model.

#### 2.3.2. Sleep Quantity, Quality, Latency, and Efficiency

This short sleep questionnaire is based on the Pittsburgh Sleep Quality Index (PSQI), which comprehensively assesses overall sleep quality and disturbances over the most recent past month [38]. While the full PSQI evaluates seven components, this version focuses on 4 selected items: sleep efficiency, sleep latency, sleep quality, and sleep duration. Sleep efficiency evaluates the ratio of time spent asleep to time in bed, categorized as good (>85%) or poor (<85%). Sleep latency measures the time taken to fall asleep, classified as good (<20 min) or delayed (>20 min). Sleep quality is subjectively rated on a 4-point scale, ranging from “very good” to “very bad”. Sleep duration is categorized based on age-specific recommendations [39]: for individuals under 65, 7–9 h is optimal, while for those 65 or older, 7–8 h is considered ideal. The reliability of the PSQI has been demonstrated with a Cronbach’s α of 0.83, indicating good internal consistency [38].

#### 2.3.3. Insomnia Severity Index (ISI)

The ISI is a self-reported questionnaire designed to evaluate the severity and impact of insomnia symptoms [40]. It consists of seven items assessing difficulties falling asleep, staying asleep, waking too early, satisfaction with sleep, daily functioning, noticeability of sleep problems, and distress caused by insomnia. Each item is scored on a scale from 0 to 4, resulting in a total score between 0 and 28. Scores classify insomnia severity as absence of insomnia (0–7), sub-threshold insomnia (8–14), moderate insomnia (15–21), or severe insomnia (22–28) [40]. The reliability of the ISI has an overall reliability coefficient (Cronbach’s α) of 0.86 [41], indicating good internal consistency, and an intraclass correlation coefficient (ICC) of 0.87 [40], reflecting strong test–retest reliability.

#### 2.3.4. Depression Anxiety Stress Scale-21 (DASS-21)

The DASS-21 is a validated self-report instrument measuring depression, anxiety, and stress symptoms experienced over the most recent past week. The 21 items are divided equally among the three scales, and responses are rated on a 4-point Likert scale. Scores for each scale are summed and doubled to determine severity, which is categorized into normal, mild, moderate, severe, or extremely severe [42].

#### 2.3.5. Short Life Satisfaction Questionnaire Lockdown (SLSQ)

The SLSQ is a modified version of the Satisfaction with Life Scale (SWLS), adapted to include three items strongly associated with emotional well-being. Previously validated and used during the COVID-19 home confinement period [43,44], the SLSQ enables individuals to make a conscious evaluative judgment of their life satisfaction using their own criteria. Participants rated their agreement with each item on a 7-point Likert scale, ranging from 1 (“Strongly disagree”) to 7 (“Strongly agree”), with total scores ranging from 3 to 21. Higher scores indicate greater life satisfaction, categorized as follows: 3 (“Extremely dissatisfied”), 4–6 (“Dissatisfied”), 7–9 (“Slightly dissatisfied”), 10–12 (“Neutral”), 13–15 (“Slightly satisfied”), 16–18 (“Satisfied”), and 19–21 (“Extremely satisfied”).

#### 2.3.6. International Physical Activity Questionnaire Short Form (IPAQ-SF)

The IPAQ-SF is a self-reported questionnaire designed to assess physical activity levels across different intensities—including vigorous, moderate, and walking activities—over the past seven days (questions 1 to 6), as well as sedentary behavior through daily sitting time (question 7). Based on responses to questions 1 through 6, the total physical activity is calculated in MET-minutes per week (Metabolic Equivalent Task), where activities are classified into three levels: low activity (<1500 MET-minutes/week), moderate activity (1500–2999 MET-minutes/week), and high activity (≥3000 MET-minutes/week). The IPAQ-SF is widely used for evaluating physical activity patterns and sedentary behavior in diverse populations and supports both clinical assessments and research studies on physical activity and health outcomes [45,46].

#### 2.3.7. Short Social Participation Questionnaire—Lockdowns (SSPQ)

The SSPQ is a short, modified version of the Social Participation Questionnaire (SPQ) designed to assess social participation behaviors during the last 12 months and previously validated and used during the COVID-19 home confinement period [43,44]. It includes 14 items, with 10 items rated on a 5-point scale from “never” to “all the time” and 4 items requiring binary “yes” or “no” responses. The total score of the SSPQ-L is calculated as the sum of the points from the 14 questions, ranging from 14 to 70. A score of 14 indicates that the participant has “never” been socially active, while scores between 15 and 28 reflect “rarely” being socially active. Scores between 29 and 42 indicate “sometimes” being socially active, scores between 43 and 56 reflect “often” being socially active, and scores between 57 and 70 represent “being socially active at all times”.

#### 2.3.8. Short Technology-Use Questionnaire—Lockdowns (STuQL)

The STuQL was developed to assess technology use for social participation, dietary practices, and physical activity. The questionnaire was previously validated and used during the COVID-19 home confinement period [43,44]. It includes three items rated on a 5-point scale from “never” to “all the time.” Scores range from 3 (minimal use) to 15 (extensive use), with intermediate categories reflecting varying levels of technology engagement.

#### 2.3.9. The MedDiet Barriers Questionnaire (MBQ)

The MBQ is a tool designed to assess barriers and limitations to adherence to the MedDiet [35,36]. The questionnaire includes 13 items exploring the presence (answered as “Yes”) or absence (answered as “No”) of various potential barriers identified in the literature. Specifically, the MBQ addresses the following: (i) barriers related to food allergies and intolerances; (ii) barriers related to cultural and/or religious limitations; (iii) barriers related to medical or health-related limitations; (iv) barriers related to individual beliefs (e.g., vegan or vegetarian diets); (v) barriers related to taste dislike; (vi) barriers related to attitudes, such as suitability, taste, restrictiveness, or food waste concerns; (vii) barriers related to social norms (e.g., food culture); (viii) barriers related to low motivation; (ix) barriers related to price unaffordability; (x) barriers related to time or effort-consuming meal preparation; (xi) barriers related to low accessibility or availability of Mediterranean food (MedFood); (xii) barriers related to a lack of knowledge and cooking skills; and (xiii) other barriers. Responses are scored as “No” = 0 and “Yes” = 1, with the total score calculated as the sum of all items, ranging from 0 to 13. A score of 0 indicates the absence of barriers, while a score of 13 reflects severe barriers to adherence.

#### 2.3.10. Additional Questions

The survey also included specific questions designed to capture geo-demographic, socio-economic, and health-related characteristics, as well as participants’ awareness of the MedDiet and lifestyle. These questions gathered detailed information on variables such as age, gender, marital status, education level, employment status, living environment (urban, suburban, or rural), country of residence, ethnicity, smoking habits, and body mass index (BMI). BMI classifications were defined according to standard WHO thresholds: underweight (<18.5 kg/m^2^), normal weight (18.5–24.9 kg/m^2^), overweight (25.0–29.9 kg/m^2^), and obese (≥30.0 kg/m^2^). Health status was assessed by categorizing participants as healthy (no known current disease), at risk of disease, or currently living with a diagnosed disease, such as cardiovascular or neurodegenerative disease. The “at risk” category was defined based on (i) the presence of two or more cardiovascular risk factors, including hypertension, dyslipidemia, high cholesterol, overweight/obesity, current smoking, or physical inactivity, or (ii) a history of cardiovascular disease (stroke, transient ischemic attack, myocardial infarction, angina pectoris, or peripheral arterial disease), or (iii) the presence of diabetes mellitus, or (iv) a combination of these conditions [47,48,49]. For analysis purposes, age was considered both as a continuous variable and a categorical variable, with participants classified into young adults (18–35 years), middle-aged adults (36–55 years), and older adults (>55 years) [50].

Awareness questions evaluated participants’ familiarity with the principles of the MedDiet.

### 2.4. Statistical Analysis

Descriptive statistics were used to summarize the distribution of MedLife adherence levels across geo-demographic and socio-economic characteristics. All statistical analyses were performed using SPSS version 25. To examine the associations between MedLife adherence and the various assessed variables, two complementary analytical approaches were adopted. First, a chi-square test of independence (χ^2^) was used to evaluate the association between MedLife adherence categories (low, moderate, and high) and categorical demographic variables. The normality of continuous variables related to consumer behaviors and psychological states was assessed using the Shapiro–Wilk test. As all variables deviated from normality, the Kruskal–Wallis test was applied to assess differences in these variables across the three MedLife adherence groups. When significant differences were identified, post hoc comparisons were conducted using the Dunn–Bonferroni test to determine pairwise group differences. Second, a series of hierarchical multiple linear regression models were conducted to explore the contribution of distinct predictor categories—including socio-demographic, psychological, behavioral, and environmental factors—to MedLife index scores. Model 1 evaluated the contribution of sociodemographic and selected health-related variables. Model 2 examined the contribution of regional and environmental factors. Model 3 focused on psychological variables, while Model 4 investigated the role of sleep-related factors. Model 5 considered physical and social activity indicators, including technology use behaviors. Model 6 assessed perceived barriers to adherence as potential predictors of MedLife scores. Subsequently, a comprehensive model (Model 7) was developed by incorporating all significant predictors identified in Models 1 through 6 to examine their combined contributions to adherence scores. Finally, a refined model (Model 8) was constructed, retaining only the significant predictors from Model 7 to achieve a more parsimonious model, while preserving explanatory power. Models’ performance were evaluated using multiple statistical criteria, including Adjusted R^2^ to assess the proportion of variance explained, standardized beta coefficients (*β*) to determine the relative strength of each predictor, and t-values and *p*-values to establish statistical significance. Semi-partial correlations (R) were examined to assess the unique contribution of each predictor to the dependent variable while controlling for the other variables in the model. The overall significance of each regression model was tested using ANOVA F-values and associated *p*-values to determine whether the predictors collectively explained a significant portion of the variance in Medlife adherence.

Significance was accepted for all analyses, a priori, at the level of *p* < 0.05.

## 3. Results

Data collected from 4010 respondents (59.5% female; mean age: 37.25 ± 15.39 years; BMI = 24.80 ± 4.85 kg/m^2^) from the MEDIET4ALL survey were analyzed.

### 3.1. Association Between Geo-Demographic, Socio-Economic, and Health Status and MedLife Adherence Levels

Table 1 presents the geo-demographic, socio-economic, and health status information categorized by the MedLife adherence levels. The analysis revealed significant associations between adherence levels and several factors. Among the countries of residence, the distribution of adherence levels varied significantly (*p* < 0.001), with Spain showing the highest proportion of high adherence (38%), followed by Italy (27%), Luxembourg (25%), and Tunisia (25%). Adherence levels also varied significantly across regions and continents (*p* < 0.001), with Mediterranean region (24%) and Europe (25%) exhibiting the highest percentage of high adherence. Ethnicity (*p* < 0.001) and living environment (*p* = 0.002) were significantly associated with adherence. White/European and Black/African/Caribbean participants demonstrated a higher proportion of high adherence (26%) compared to Turks (16%) and Asians (17%). Participants from urban environments exhibited the highest proportion of high adherence (23%).

Age (*p* = 0.037) and BMI (*p* = 0.002) were also significantly associated with adherence. Elderly participants (>55 years) showed the highest proportion of high adherence (25%), as did individuals with a normal BMI, 18.5 to 24.9 kg/m^2^ (23%).

Education, MedLife awareness, and employment status were strongly associated with adherence (*p*-values ranging from 0.001 to *p* < 0.001). Participants with a Master’s or Doctorate degree, those aware of MedLife principles, and individuals with stable economic situations (e.g., employed or retired) demonstrated the highest proportion of high adherence (23–29%). In contrast, individuals with no formal schooling, no awareness of MedDiet principles, or those who were unemployed exhibited the lowest adherence levels (13–18%). Marital status was also a significant factor (*p* = 0.007), with married individuals living as a couple exhibiting the highest proportion of high adherence (24%), whereas widowed, divorced, and separated participants showed the lowest (18%).

Health status (*p* = 0.004) was another significant factor, with healthy individuals showing the highest proportion of high adherence (23%), compared to those categorized as “with disease” (17%). Smoking habits, however, showed comparable proportions of high adherence among smokers and non-smokers. Gender was not significantly associated with adherence levels.

### 3.2. Potential Associations Between MedLife Adherence Levels and Consumer Behaviors: Findings of the Kruskal–Wallis Test

**Figure 1:**
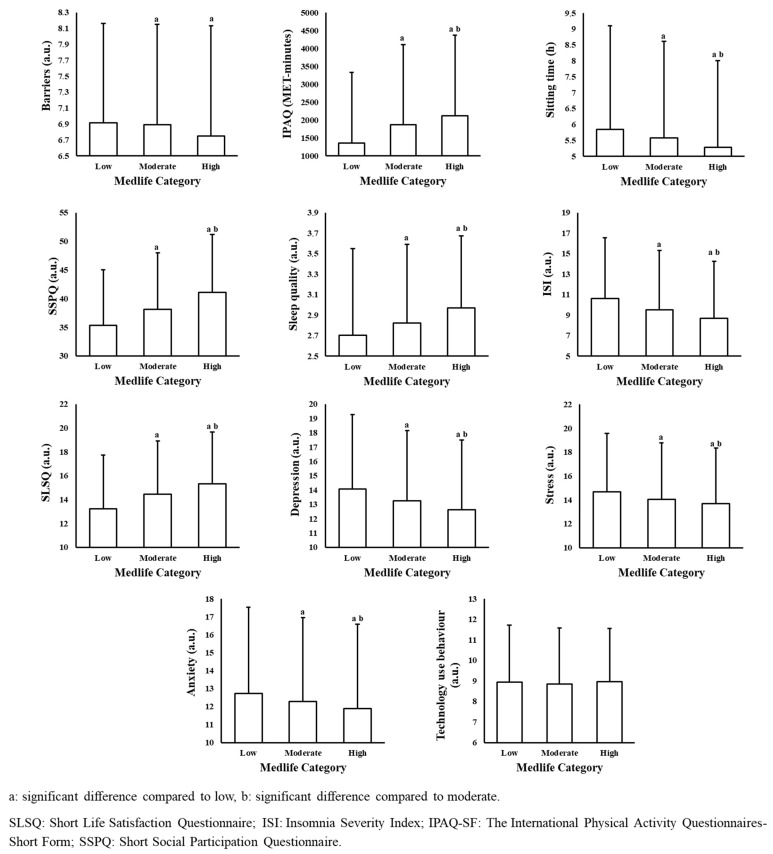
Potential associations between MedLife adherence levels and consumer behaviors.

Figure 1 illustrates the associations between MedLife adherence levels and various consumer behaviors. A significant difference was observed in the total barriers score (H = 52.8, *p* < 0.001), with participants in the high- and moderate-adherence groups reporting significantly lower perceived barriers compared to those in the low-adherence group (Z = 5.82, *p* < 0.001 and Z = 5.58, *p* < 0.001, respectively). The MedLife adherence level was also significantly associated with physical activity (IPAQ), sitting time, and social participation (H = 138.93, 15.9, and 174.97, respectively; all *p* < 0.001). Participants with high adherence reported significantly greater physical activity and social participation, as well as significantly less sitting time, compared to those with moderate and low adherence (all *p* < 0.001). In addition, participants with moderate adherence reported higher physical activity and social participation levels than those with low adherence (Z = 8.6 and Z = 7.78, respectively; both *p* < 0.001), although no significant difference in sitting time was observed between moderate- and low-adherence groups (Z = 2.10, *p* = 0.107).

Significant associations were also found between MedLife adherence levels and both sleep quality and insomnia severity (H = 51.27 and 54.46, respectively; *p* < 0.001 for both). Participants with high adherence reported significantly better sleep quality and lower insomnia severity compared to those with moderate (Z = 4.51 and Z = 7.25, respectively) and low adherence (Z = 7.16 and Z = 4.79, respectively; all *p* < 0.001). Similarly, participants with moderate adherence demonstrated better sleep quality (Z = 3.57, *p* = 0.001) and lower insomnia severity (Z = 3.52, *p* < 0.001) compared to those with low adherence.

Life satisfaction scores were significantly different across adherence levels (H = 119.46, *p* < 0.001). Participants in the high-adherence group reported greater life satisfaction than those in the moderate- (Z = −10.68, *p* < 0.001) and low-adherence groups (Z = −7.36, *p* < 0.001), and those with moderate adherence scored higher than those with low adherence (Z = −4.90, *p* < 0.001).

Significant differences in psychological outcomes were also observed. Depression (H = 48.24), anxiety (H = 20.18), and stress (H = 26.47) scores varied across adherence levels (all *p* < 0.001). Participants with high adherence reported significantly lower levels of depression (Z = 6.85 and Z = 4.41), anxiety (Z = 4.48 and Z = 2.50), and stress (Z = 4.94 and Z = 3.71) compared to both moderate- and low-adherence groups (all *p* ≤ 0.012). Additionally, those with moderate adherence showed significantly lower levels of depression (Z = 3.43, *p* < 0.001), anxiety (Z = 2.59, *p* = 0.015), and stress (Z = 2.00, *p* = 0.045) compared to the low-adherence group.

No significant association was found between the MedLife adherence level and total technology use behaviors (H = 1.46, *p* = 0.483).

### 3.3. Potential Predictors of Adherence to the Mediterranean Lifestyle: Findings from the Hierarchical Multiple Linear Regression Models

Table 2 presents the results of six predictive models, each evaluating the potential predictive role of different factors on MedLife adherence. The explained variance across the models varied, as indicated by the Adjusted R^2^ values. Model 6, which assessed perceived barriers, demonstrated the highest explanatory power (Adjusted R^2^ = 0.084), with MedDiet awareness showing the strongest positive association (β = 0.248), alongside individual beliefs, taste dislike, social norms, low motivation, price unaffordability, time/effort-consuming and low accessibilities and availability barriers, all showing significant negative associations. Model 5, focusing on physical and social activities, accounted for moderate variance (Adjusted R^2^ = 0.073). Social participation (β = 0.219) emerged as the strongest predictor, followed by physical activity, both with significant positive associations, while sitting time showed a significant negative association. Psychological factors (Model 3, Adjusted R^2^ = 0.035), sleep patterns (Model 4, Adjusted R^2^ = 0.027), and demographic factors (Model 1, Adjusted R^2^ = 0.027) showed lower explanatory power, though life satisfaction (SLSQ-L, β = 0.168), age (β = 0.126), sleep quality (β = 0.089), and education (β = 0.088) were the most notable predictors with significant positive association, while BMI (β = −0.074) and the insomnia index (β = −0.068) showed a significant negative association. Geographic factors (Model 2, Adjusted R^2^ = 0.014) explained the least variance, with region (β = 0.094) emerging as a key significant contributor, followed by continent and living environment. Non-significant variables included gender, marital status, employment, ethnicity, country of living, depression, anxiety, stress, sleep quantity, latency and efficiency, technology use, and some barriers such as allergies/intolerances, cultural, medical, attitude, and lack of knowledge-related ones.

As a continuation of the analysis in Table 2, Table 3 presents the results of two comprehensive predictive models incorporating demographic, geographic, psychological, sleep, physical, social engagement variables, and barrier factors. Model 7 evaluates the predictive role of all significant factors from Models 1 to 6 in Table 2 on MedLife adherence. Model 8 represents a final refined version, including only the significant factors identified in the comprehensive Model 7. The refined regression model (Model 8) explains 17.9% of the variance in adherence to MedLife (Adjusted R^2^ = 0.179). Among the predictors, MedDiet awareness (β = 0.206) emerged as the strongest positive predictor, followed by social participation (β = 0.194) and physical activity (β = 0.096). Life satisfaction, sleep quality, and age (β ranging from 0.071 to 0.093) also contributed positively, highlighting the importance of adequate rest, overall satisfaction, and the greater likelihood of adherence among older individuals. Education and living in the Mediterranean region had smaller but significant positive associations with MedLife scores (β ranging from 0.049 to 0.058), reflecting the supportive roles of higher education levels and the regional context.

Conversely, factors such as prolonged sitting time, living environment, and barriers—including individual beliefs, taste dislike, low motivation, price unaffordability, time/effort-consuming preparation, and low accessibility or availability of MedFood—were negatively associated with adherence, with standardized beta coefficients ranging from −0.036 to −0.067. These findings indicate that sedentary behavior, multiple perceived barriers, and differences between urban and rural settings may contribute to lower MedLife adherence, with urban environments appearing slightly more favorable for MedLife adherence than rural ones.

## 4. Discussion

The primary aim of this study was to comprehensively analyze geo-demographic, socio-economic, psychological, behavioral, and barrier-based factors associated with and potentially contributing to MedLife adherence across young, middle-aged, and older adults from ten Mediterranean and neighboring countries. Using validated MedDiet adherence scores aligned with the MEDIET Pyramid and data collected through the MEDIET4ALL multilingual e-survey—which included responses from over 4000 participants—this study aimed to identify the significant predictors of adherence and explore their interactions. The findings revealed that adherence levels were significantly associated with various geo-demographic and socio-economic factors. Behavioral and psychological variables differ markedly across adherence levels. Most notably, the refined comprehensive regression models highlighted several significant predictors of MedLife adherence. Positive contributions included greater awareness and education, participation in physical and social activities, good sleep quality, older age, and higher life satisfaction. Conversely, adherence was negatively associated with sedentary behavior, and barriers such as individual beliefs, low motivation, taste dislike, price unaffordability, limited availability, and the time-consuming nature of preparing MedFood. Region and living environment also emerged as significant contributors, although their associations with adherence were relatively smaller.

In terms of geo-demographic and socio-economic factors, the main findings revealed that the prevalence of low-, medium-, and high-adherence responders varied significantly by country, region, continent, living environment, and ethnicity. Participants from Spain, those living in Europe, in the Mediterranean region, and those in urban environments exhibited the highest proportion of “high” adherents. Age, BMI, education, MedLife awareness, employment status, marital status, and health status were also significantly associated with adherence. Older participants (>55 years), those with a normal BMI (18.5 to 24.9 kg/m^2^), individuals with higher educational attainment and awareness of the MedLife principles, as well as married individuals living as a couple, demonstrated the highest proportion of “high” adherence. Awareness, education, and the region of living showed significant contributions to MedLife adherence across various regression models, with awareness emerging as the strongest predictor. However, gender showed a limited association with adherence, with comparable adherence proportions between these groups.

These findings align with prior research emphasizing the critical roles of education, awareness, employment, marital status, and health status in adherence to MedLife [22,23,24]. Adults with higher education, greater awareness, and stable employment status typically exhibit greater adherence to MedLife principles, largely due to increased access to resources and higher levels of nutritional literacy [51,52,53,54]. Individuals with greater knowledge of MedDiet principles have been suggested to be more likely to adhere to its guidelines. This is supported by Elmskini et al. [55], who demonstrated a positive correlation between MedDiet adherence scores and nutrition knowledge scores. Living in the Mediterranean region also appears to be associated with higher adherence to MedLife. This is supported by a recent regional comparative study [35], which revealed distinct dietary patterns, with Mediterranean participants showing higher scores in Block 1 (Mediterranean Food Consumption) of the MedLife index—indicating stronger adherence to recommended intakes of traditional MedDiet components such as legumes and fish. Furthermore, participants scored higher in Block 2 (Mediterranean Dietary Habits), with a greater proportion adhering to recommendations on water intake, limiting salt in meals, reducing snack consumption, and avoiding nibbling between meals. However, these regional differences should be interpreted with caution, as potential confounding factors—such as differences in sampling strategies, population characteristics, or unmeasured socio-cultural influences—may have influenced the observed results.

Similarly, education campaigns, such as those highlighted by Sotos-Prieto et al. [33], have been shown to significantly improve adherence levels by promoting the health benefits of the MedDiet and lifestyle. These campaigns enhance food and nutrition literacy, empowering individuals to understand the long-term benefits of the MedDiet and prioritize its adoption for themselves and their families [56]. Moreover, previous research consistently links parental education to higher adherence to the MedDiet, underscoring the intergenerational benefits of nutrition literacy [52,53,54]. Parents with a strong understanding of the MedDiet and its health advantages are more likely to instill these dietary habits in their family members [56]. Conversely, a lack of awareness about the diet’s health impacts may lead to the underappreciation and poor adoption of healthy eating patterns. The regression analysis in the present study further supports these observations, identifying education and awareness as significant positive predictors of adherence, with awareness emerging as the strongest predictor across models. These findings underscore the critical role of education and awareness campaigns in promoting adherence by empowering individuals to make informed dietary choices and incorporate key MedFood, such as olive oil, nuts, and fresh vegetables, into their diets. To further enhance MedLife adherence, policymakers should consider implementing community-based campaigns and public health initiatives that improve nutritional literacy, focusing on sustainable and culturally tailored strategies to foster healthy eating habits across diverse demographic groups.

The higher proportion of individuals with ‘high’ MedLife adherence among those with normal weight and better overall health, compared to those who are overweight, obese, or at risk of or already diagnosed with chronic diseases, further supports the well-established health benefits of adhering to the MedDiet and MedLife principles [1]. This adherence has been shown to reduce the risk of cardiovascular and neurodegenerative diseases, as well as obesity [7,8,9]. Furthermore, the higher adherence levels observed among married individuals living as a couple, compared to those who are widowed, divorced, or separated, aligns with previous findings emphasizing the stabilizing influence of shared meal preparation and consumption on dietary habits [57]. Shared meals were shown to be associated with improved diet quality, including an increased consumption of fruits and vegetables and a reduced intake of high-calorie, low-nutrient foods [58]. These patterns underscore the critical role that social and familial structures play in fostering adherence to healthy dietary patterns, including MedDiet. Notably, social involvement, such as engagement with family, is an integral component of the modern MedDiet pyramid [1,2].

While the observed higher adherence in countries such as Spain, followed by Italy and Tunisia, as well as in the broader Mediterranean region compared to non-Mediterranean regions aligns with the findings of Keys et al. [59]—which highlighted proximity to fresh produce and cultural dietary norms as key contributors—the fact that high adherence levels did not exceed 24% even within Mediterranean countries underscores a concerning decline in MedDiet adherence observed over recent decades, both globally and within its region of origin [12,13]. This decline is largely attributed to the rising consumption of ultra-processed foods (UPFDs) [14,17], which have been shown to contribute to over 50% of nutrient intake in several EUR-MED countries [18], and the proliferation of Western dietary patterns [19]. Otherwise, it is generally accepted that adherence to the MedDiet and its lifestyle tends to differ between rural and urban populations, with research generally indicating higher adherence levels in rural areas. A study conducted in eastern Sicily by Grosso et al. [60] found that rural residents scored slightly higher on adherence to the MedDiet compared to their urban counterparts, likely due to the preservation of traditional dietary patterns. Rural populations often have greater access to fresh, locally produced foods, including fruits, vegetables, legumes, and olive oil, which are staples of the MedDiet [1,2]. Additionally, rural communities are more likely to maintain cultural and familial practices that align with the principles of the MedDiet, as traditional eating habits are less influenced by Westernized, convenience-driven diets [23]. In contrast, urban environments are typically associated with a higher availability of UPFs and fast-food outlets, contributing to dietary shifts that diverge from the MedDiet [45]. However, the present findings revealed a reversed trend, with urban respondents exhibiting a slightly higher prevalence of high adherence (23% vs. 20%) and a lower prevalence of low adherence (29% vs. 36%) compared to rural respondents. This finding may reflect the significant contribution of education and awareness on MedLife adherence, as urban populations often have greater access to health education and awareness campaigns, which may mitigate the negative effects of dietary shifts. In our study, a higher proportion of urban respondents held Master’s or PhD degrees compared to their rural counterparts (24% vs. 16%) and demonstrated higher awareness scores, further supporting this interpretation. Additionally, the increasing globalization and urbanization of food systems appear to be eroding traditional rural dietary patterns over time [55]. These findings highlight the importance of promoting MedDiet adherence through targeted strategies that address the unique challenges and opportunities presented by both rural and urban environments. Tailored interventions should emphasize the preservation of traditional dietary practices in rural areas and leverage educational and awareness initiatives in both urban and rural settings to sustain and enhance adherence to the MedLifestyle.

Regarding the age factor, the present findings contradict previous studies that reported a decline in adherence to the MedDiet with age [25,26,27] and instead support those indicating an increase in adherence among older adults. This trend is likely driven by the higher prevalence of nutrition-related disorders in older populations, which often require dietary modifications aligning with MedDiet principles [28]. Additionally, increased health awareness and more stable eating patterns among older adults further contribute to their higher adherence levels [22].

Similarly, regarding gender differences, our findings contradict studies suggesting better adherence among women [29] but are consistent with those reporting no significant differences between men and women [30]. The absence of a significant gender-based association with MedLife adherence in our study may be partially explained by the comparable levels of education and awareness among male and female respondents. For instance, the proportion of participants holding a Master’s degree or PhD was 22% among men and 24% among women. These factors—identified as potential contributors to MedLife adherence in our meta-regression models—may have attenuated any gender-related differences in adherence.

In terms of behavioral factors influencing MedLife adherence, the present study revealed that highly adherent responders exhibited significantly higher scores for physical activity, social participation, sleep quality, and life satisfaction compared to moderately and lowly adherent individuals. Similarly, moderately adherent responders scored higher in these domains compared to those with low adherence. These findings suggest that enhanced life satisfaction, along with a more physically and socially active lifestyle, is associated with higher adherence to the MedLife principles. Conversely, behaviors such as av prolonged sitting time, insomnia, and perceived psychological disorders—including depression, stress, and anxiety—were significantly lower among individuals with higher MedLife adherence. This indicates that adherence to MedLife principles may contribute to reduced sedentary behavior and psychological distress, further supporting and enriching the growing body of evidence on the holistic health benefits of adhering to the MedLifestyle [61].

Regular physical activity (PA) is widely recognized as a critical factor in preventing non-communicable diseases (NCDs) and mitigating risk factors such as overweight and obesity while promoting overall physical and mental well-being [62]. In contrast, a sedentary lifestyle is a well-documented risk factor for a range of health issues, including obesity, cardiovascular diseases, and cognitive decline [63]. Recent findings from the Global Burden of Disease study indicate that the contribution of low physical activity to deaths and disability-adjusted life years (DALYs) has increased by over 80% since 1990, with approximately 1 million deaths and 16 million DALYs attributed to physical inactivity in 2019 [64]. Reducing physical inactivity by just 10% could prevent more than 530,000 deaths annually, while a 25% reduction could save over 1.3 million lives [65]. Beyond its health benefits, physical activity offers significant economic advantages. According to the World Health Organization (WHO), physical inactivity costs the global economy over USD 54 billion annually in direct healthcare expenses and an additional USD 14 billion in lost productivity [66]. Promoting physical activity at a population level could alleviate these financial burdens, underscoring its importance not only for individual health but also for global economic sustainability.

The positive association between physical activity and MedLife adherence observed in our study is supported by higher IPAQ scores and lower sitting time among highly adherent responders. This relationship is further reinforced by the significant positive contribution of physical activity and the significant negative association of sitting time on MedLife adherence, as revealed across various regression models. These findings are consistent with those of García-Hermoso et al. [67], who reported that individuals adhering to the MedDiet are more likely to engage in regular physical activity. Similarly, our observation of reduced sitting time among highly adherent individuals and its significant negative association with MedLife adherence aligns with evidence from Mendes et al. [68] and Júdice et al. [69], who found that sedentary behavior is inversely correlated with adherence to MedLife.

The significant associations of both physical activity and reduced sedentary behavior with MedLife scores further highlight their synergistic role in promoting MedLife adherence. The integration of physical activity with MedDietary practices [1,2] is thereby suggested to amplify its health and economic benefits, making it a cornerstone of both individual and public health strategies.

Beyond physical activity and healthy dietary patterns, social inclusion—a critical component of Active Healthy Living and particularly the MedLifestyle—is essential across the lifespan. Social isolation and loneliness are well-documented risk factors for numerous physical and mental health conditions, including depression, cognitive decline, and even premature mortality [70]. Recent data from the European Commission indicate that over 75 million European adults meet with family or friends no more than once a month, and approximately 7% report frequent loneliness [71].

In our study, highly adherent individuals reported significantly greater social participation. Across various regression models, social participation emerged as the second strongest positive contributor to MedLife adherence, following awareness. These findings are consistent with Bonaccio et al. [72], who emphasized the integral social dimension of the MedDiet, which traditionally includes shared meals and family gatherings. Social involvement is also highlighted in the modern MedDiet pyramid as a key factor supporting dietary adherence [1,2]. Engaging in social activities strengthens cultural and familial bonds while encouraging adherence to healthier dietary practices through shared meal preparation and consumption [73].

The positive contribution of social participation aligns with its role in reinforcing cultural dietary practices and facilitating shared experiences, both of which are foundational to MedLife. Bonaccio et al. [72] underscored how socialization fosters adherence by making healthy eating a collective, culturally rooted activity.

Moreover, the combined strong contributions of physical activity and social participation on MedLife adherence, as revealed by our regression models, support findings from Psaltopoulou et al. [9], who emphasized the importance of community engagement and active lifestyles in sustaining MedDiet patterns. Together, these elements highlight the holistic nature of MedLife, where dietary, physical, and social behaviors synergistically contribute to health and well-being.

Furthermore, the present study demonstrated higher sleep quality and lower insomnia severity among highly adherent individuals, with sleep quality showing a significant positive contribution to MedLife adherence across various regression models. These findings align with prior research linking the MedDiet to better sleep health. Godos et al. [74] and St-Onge et al. [75] highlighted the role of the nutrient composition of the MedDiet—rich in antioxidants, omega-3 fatty acids, and tryptophan-containing foods [72]—in improving sleep duration and quality and reducing insomnia symptoms. Similarly, Godos et al. [76] found that adherence to the MedDiet is associated with a lower risk of insomnia, likely due to its anti-inflammatory properties and its role in promoting stable glucose levels, which are critical for regulating sleep patterns.

In terms of psychological health, our findings revealed significantly lower levels of depression, stress, and anxiety among highly adherent individuals. These results are consistent with meta-analyses by Psaltopoulou et al. [9] and Lassale et al. [77], which demonstrated that adherence to the MedDiet is inversely associated with depression and anxiety. The MedDiet’s high content of anti-inflammatory and antioxidant nutrients, including omega-3 fatty acids, polyphenols, and vitamins, has been shown to modulate neuroinflammation and oxidative stress [72], which are mechanisms linked to psychological disorders. Additionally, its ability to regulate the gut–brain axis and enhance serotonin synthesis provides further support for its role in enhancing brain functions and reducing mental health risks [78,79]. Furthermore, the role of high social participation, observed among highly adherent responders, is well-documented in alleviating mental health problems, as noted by Holt-Lunstad et al. [70], further emphasizing the interconnected benefits of MedLife.

Similarly, life satisfaction was significantly higher among individuals with greater adherence to MedLife in our study. Comprehensive regression models revealed a strong positive association, consistent with findings from Grao-Cruces et al. [80,81] and Zaragoza-Martí et al. [32]. These studies reported a positive correlation between adherence to the MedDiet and life satisfaction, particularly among older women [32] and adolescents [80,81]. The holistic nature of MedLife—emphasizing fresh, nutrient-dense foods, regular physical activity, and strong social connections—appears to collectively enhance well-being and improve overall quality of life.

In addition to socio-economic, geographical, behavioral, and psychological factors, perceived barriers and enablers such as motivation have been extensively investigated in nutrition science [81], given their pivotal role in triggering behavior change and shaping adherence [82]. For instance, previous studies have shown that individuals with higher health motivation are more likely to adopt MedLife practices [83,84]. The present results across various regression models align with these findings, with the refined comprehensive models revealing significant negative associations between MedLife scores and barriers such as “low motivation”, “time/effort-consuming” preparation of MedFood, and “taste dislike” alongside food choices based on “individual beliefs” (e.g., vegan or vegetarian diets). These findings underscore the contribution of personal values and perceived effort on adherence, highlighting that unfavorable taste perceptions, the labor-intensive nature of traditional Mediterranean recipes, and low motivation may hinder adherence. Addressing these barriers through targeted education and behavior modification strategies is critical for improving adherence. Additionally, this study identified “price unaffordability” and “limited accessibility and availability” as significant barriers negatively associated with adherence to MedLife. These findings are consistent with prior research indicating that high food costs and limited access to healthy options are substantial barriers to dietary adherence, particularly in urban–rural settings and low-income populations where the economic effort required to be able to afford the MedDiet is higher [85,86]. Addressing these issues will require systemic solutions that focus on improving the availability, accessibility, and affordability of MedFood items. Governments and policymakers could collaborate with food suppliers to ensure that essential MedFoods are stocked in major residential districts and that delivery options are widely available. Subsidizing healthy MedFood items could further alleviate financial barriers, making these options more accessible to diverse populations.

The findings—particularly the significant negative contribution of the ‘time/effort-consuming’ barrier—underscore the importance of modernizing traditional Mediterranean recipes to better align with contemporary lifestyles. Developing healthier, quicker, and less labor-intensive versions that retain the authentic flavors of Mediterranean cuisine (e.g., using herbs instead of high-calorie sauces or honey in place of sugar) may help overcome time and effort-related barriers to adherence. Furthermore, comprehensive promotion strategies for MedLife should leverage its holistic characteristics by focusing not only on dietary interventions but also on enhancing social participation, physical activity, and psychological well-being.

Overall, the updated results—particularly those from the refined comprehensive regression model—offer valuable insights into the key factors associated with adherence to MedLife. While the predictive power of the models was modest, the findings consistently highlight the relevance of MedDiet awareness, behavioral factors such as social participation and physical activity, psychological aspects such as life satisfaction, and barriers including taste preferences, low motivation, price unaffordability, limited accessibility, and time-consuming preparation. These results underscore the need for integrated, multi-level strategies—such as awareness campaigns, community-based initiatives, and targeted behavioral and policy interventions—to support adherence and promote the broader health benefits of the Mediterranean lifestyle.

### 4.1. Strengths, Limitations, and Perspectives

The findings of this study should be interpreted considering its strengths and limitations. A notable strength is its multinational design, encompassing ten Mediterranean and neighboring countries. The study employed a consistent methodology and was conducted within the same time frame, underscoring its comparability across regions. Additionally, the large sample size of over 4000 participants enhances the credibility and statistical power of the findings.

By assessing socio-economic, geo-demographic, behavioral, psychological, and potential barriers to adherence to MedLife and utilizing validated MedDiet adherence scores aligned with the MEDIET Pyramid, this study offers a comprehensive understanding of the factors influencing dietary adherence in the MedDiet.

However, there are some limitations to consider. First, as a cross-sectional study, it cannot establish causal relationships between the identified factors and MedLife adherence. Second, the reliance on self-reported data collected through surveys and questionnaires introduces the possibility of bias and inaccuracies. Third, this study included a relatively small number of participants over 55 years old (*n* = 669) and a low prevalence of obesity (*n* = 41), which may limit the generalizability of the findings to older adults and individuals with obesity, calling for cautious interpretation of the related results. Fourth, the online data collection method may have excluded individuals with lower educational levels or limited digital literacy, potentially reducing the representativeness of the sample. Fifth, while this study has identified associations between various socio-demographic, behavioral, and psychological factors and dietary adherence, the underlying mechanisms remain unclear. The discussion of these mechanisms relies on scientific speculation based on the existing literature.

Future research should aim to investigate the mechanistic pathways linking behavioral and psychological factors to MedLife adherence, including the influence of motivation, health literacy, and perceived barriers. Longitudinal studies employing objective measurement tools—such as wearable devices for physical activity, digital dietary tracking, or ecological momentary assessment—are essential for confirming causality and capturing temporal variations in adherence behaviors. Additionally, exploring culturally adapted, country-specific strategies to promote MedLife across diverse populations will be valuable for tailoring effective interventions.

Importantly, qualitative research approaches offer a promising complementary avenue to deepen our understanding of the lived experiences, cultural meanings, and social processes that shape dietary practices and lifestyle adherence. Cross-national comparative studies using ethnographic methods, in-depth interviews, focus groups, or content analysis of food marketing campaigns could provide context-rich insights into how MedLife is perceived, practiced, or resisted across socio-cultural settings. Such work would allow researchers to unpack how identity, values, and symbolic meanings associated with food and health behaviors vary across countries. Drawing on foundational works such as Deborah Lupton’s *Food, the Body and the Self* could further illuminate the psychosocial dimensions of MedLife adherence and support the development of more grounded, socially informed interventions.

### 4.2. Practical Applications

The findings underscore the critical need for comprehensive interventions and a holistic approach to promote adherence to MedLife and address the declining trend in adherence. Policymakers and public health organizations should implement educational campaigns to enhance awareness of the MedDiet’s health benefits, focusing on culturally tailored, sustainable strategies to reach diverse populations. Social and community-based programs, such as family-based meal-sharing initiatives and community cooking workshops, can reinforce traditional practices and foster social participation, a key contributor to adherence.

Integrating physical activity promotion into these initiatives is essential, as regular activity and reduced sedentary behavior synergize with dietary adherence to amplify health benefits. Equally important is addressing barriers such as unfavorable taste perceptions and the time-intensive nature of traditional recipes. Developing modern, simplified recipes that preserve the authentic flavors of Mediterranean cuisine while incorporating healthier and more accessible options can significantly enhance adherence to MedLife. Holistic initiatives and research and development projects—such as MEDIET4ALL, DELICIOUS, SWITCHtoHEALTHY, PROMEDLIFE, and others—should be further encouraged through dedicated funding programs like PRIMA. Greater emphasis should be placed on fostering synergies among these initiatives, ensuring the sustainability of their approaches, and aligning their efforts to create impactful and lasting outcomes.

To ensure equitable access, governments and food suppliers should collaborate to improve the availability, affordability, and accessibility of MedFood staples, particularly in urban and underserved areas. Future research should focus on longitudinal studies to explore causal pathways and identify effective, country-specific strategies to sustain and enhance MedLife adherence. By addressing these areas, stakeholders can effectively integrate MedLife into public health policies, maximizing its potential to improve health outcomes worldwide.

## 5. Conclusions

This study provides a comprehensive, multinational analysis of the factors associated with adherence to the Mediterranean lifestyle (MedLife). The findings indicate that adherence is shaped by a complex interplay of geo-demographic, socio-economic, behavioral, psychological, and barrier-related factors. Positive contributors include a greater awareness of MedLife principles, higher educational attainment, participation in physical and social activities, better sleep quality, and higher life satisfaction. In contrast, factors such as sedentary behavior, unfavorable taste preferences, low motivation, price unaffordability, the time-consuming nature of preparing MedFood, and limited availability were negatively associated with adherence.

While the predictive power of the regression models was modest, the consistency of significant associations across multiple domains highlights the multifaceted nature of MedLife adherence. These findings underscore the importance of considering a broad range of interrelated factors when designing interventions. Future studies aiming to enhance predictive accuracy should explore the inclusion of additional explanatory variables—such as cultural attitudes, food literacy, and neighborhood food environments—and consider employing longitudinal and mixed-method designs to better capture dynamic, contextual, and potentially causal relationships.

Notably, the overall prevalence of high MedLife adherence did not exceed one-quarter of participants, with only around 22% demonstrating high adherence. This low prevalence reinforces concerns about the global decline in traditional dietary patterns, even within Mediterranean regions. The increasing consumption of ultra-processed foods (UPFs) and the spread of Western dietary habits are major contributors to this trend. These findings highlight an urgent need for multi-level, culturally sensitive interventions to preserve and promote MedLife adherence as a model for sustainable health and well-being worldwide.

## Figures and Tables

**Table 1:** Geo-demographic, socio-economic, and health status information categorized by MedLife adherence level.

Variables	Low	Medium	High	Total	χ^2^	*df*	*p*
Country of living							
*Algeria*	49 (34%)	71 (49%)	26 (17%)	146	113.93	18	<0.001
*France*	171 (32%)	239 (45%)	123 (23%)	533			
*Germany*	223 (36%)	279 (45%)	114 (19%)	616			
*Italy*	212 (30%)	304 (43%)	195 (27%)	711			
*Luxembourg*	21 (18%)	67 (57%)	30 (25%)	118			
*Tunisia*	42 (25%)	86 (50%)	42 (25%)	170			
*Spain*	50 (18%)	123 (44%)	105 (38%)	278			
*Morocco*	56 (35%)	72 (45%)	32 (20%)	160			
*Turkey*	177 (30%)	326 (55%)	93 (15%)	596			
*Jordan*	247 (36%)	308 (45%)	127 (19%)	682			
Region							
*Mediterranean*	757 (29%)	1221 (47%)	616 (24%)	2594	17.83	2	<0.001
*Non-Mediterranean*	491 (35%)	654 (46%)	271 (19%)	1416			
Continent							
*Europe*	677 (30%)	1012 (45%)	567 (25%)	2256	30.22	4	<0.001
*Asia*	424 (33%)	634 (50%)	220 (17%)	1278			
*Africa*	147 (31%)	229 (48%)	100 (21%)	476			
Ethnicity							
*Prefer not to say*	65 (32%)	92 (46%)	44 (22%)	201	41.86	14	<0.001
*Black/African*/*Caribbean*	34 (27%)	58 (47%)	33 (26%)	125			
*Latin American*/*Hispanic*	18 (29%)	25 (40%)	19 (31%)	62			
*White*/*European*	559 (29%)	855 (45%)	486 (26%)	1940			
*Asian*	38 (35%)	53 (48%)	19 (17%)	110			
*Middle Eastern*/*Arab*	300 (33%)	429 (47%)	177 (20%)	906			
*Turks*	166 (30%)	294 (54%)	90 (16%)	550			
*Other*	28 (24%)	69 (60%)	19 (16%)	116			
Living environment							
*Urban environment*	773 (29%)	1271 (48%)	614 (23%)	2658	16.57	4	0.002
*Suburban environment*	248 (34%)	330 (46%)	148 (20%)	726			
*Rural environment*	227 (36%)	274 (44%)	125 (20%)	626			
Gender							
*Male*	495 (30%)	755 (47%)	375 (23%)	1625	1.57	2	0.457
*Female*	753 (32%)	1120 (47%)	512 (21%)	2385			
Age (years)							
*18–35*	711 (33%)	1004 (46%)	455 (21%)	2170	10.19	4	0.037
*36–55*	358 (30%)	546 (47%)	267 (23%)	1171			
*>55*	179 (27%)	325 (48%)	165 (25%)	669			
Body mass index (BMI)							
*Underweight*	74 (35%)	97 (47%)	38 (18%)	209	21.44	6	0.002
*Normal weight*	617 (29%)	1031 (48%)	498 (23%)	2146			
*Overweight*	535 (33%)	735 (46%)	344 (21%)	1614			
*Obesity*	22 (54%)	12 (29%)	7 (17%)	41			
Education							
*No schooling completed*	78 (37%)	93 (45%)	37 (18%)	208	47.09	6	<0.001
*High school graduate, diploma, or the equivalent/Professional degree*	483 (34%)	665 (46%)	296 (20%)	1444			
*Bachelor’s degree*	476 (33%)	683 (47%)	296 (20%)	1455			
*Master–Doctorate degree*	211 (23%)	434 (48%)	258 (29%)	903			
MedLife awareness							
*Aware*	501 (23%)	1042 (48%)	642 (29%)	2185	218.92	2	<0.001
*Not aware*	747 (41%)	833 (46%)	245 (13%)	1825			
Marital status							
*Single*	631 (32%)	931 (47%)	411 (21%)	1973	14.23	4	0.007
*Married living as a couple*	507 (29%)	817 (47%)	422 (24%)	1746			
*Widowed, divorced, separated*	110 (38%)	127 (44%)	54 (18%)	291			
Employment							
*Employed*	609 (30%)	948 (47%)	476 (23%)	2033	25.78	8	0.001
*Unemployed*	181 (39%)	203 (43%)	83 (18%)	467			
*Student*	322 (31%)	480 (47%)	225 (22%)	1027			
*Retired*	87 (26%)	170 (50%)	82 (24%)	339			
*Uncategorized*	49 (34%)	74 (51%)	21 (15%)	144			
Health status							
*Healthy*	895 (30%)	1419 (47%)	700 (23%)	3014	15.67	4	0.004
*At risk*	248 (35%)	322 (45%)	138 (20%)	708			
*With diseases*	105 (46%)	134 (47%)	49 (17%)	288			
Smoking							
*Cigarettes smokers*	287 (36%)	335 (42%)	173 (22%)	795	12.53	4	0.014
*Shisha smokers*	61 (29%)	101 (48%)	47 (23%)	209			
*Non-smokers*	900 (30%)	1439 (48%)	667 (22%)	3006			

**Table 2:** Potential predictive role of geo-demographic, psychological, and behavioral factors in MedLife adherence.

		UC		SC	t	Sig	R	SEE	Adj. R^2^	ANOVA	
		b	SE	β						F	*p*-Value
Model 1	(Constant)	12.943	0.515		25.146	<0.001		3.11	0.027	12.4	<0.001
	Age	0.026	0.004	0.126	6.138	<0.001	0.061				
	BMI	−0.048	0.011	−0.074	−4.436	<0.001	−0.065				
	Gender	−0.136	0.102	−0.021	−1.325	0.185	−0.019				
	Marital status	−0.169	0.097	−0.033	−1.736	0.083	0.006				
	Employment	−0.023	0.043	−0.009	−0.539	0.59	−0.019				
	Health	0.353	0.087	0.067	4.052	<0.001	0.066				
	Ethnicity	−0.036	0.032	−0.018	−1.121	0.262	−0.04				
	Education	0.212	0.039	0.088	5.462	<0.001	0.09				
	Smoking	0.123	0.063	0.031	1.968	0.049	0.035				
Model 2	(Constant)	13.766	0.260		52.968	<0.001		3.13	0.014	15.43	<0.001
	Country of living	0.032	0.020	0.031	1.588	0.112	−0.011				
	Region	0.617	0.108	0.094	5.711	<0.001	0.083				
	Continent	−0.343	0.084	−0.076	−4.074	<0.001	−0.045				
	Living environment	−0.280	0.068	−0.067	−4.116	<0.001	−0.066				
Model 3	(Constant)	12.659	0.270		46.843	<0.001		3.09	0.035	36.97	<0.001
	Depression	−0.032	0.018	−0.051	−1.803	0.071	−0.106				
	Anxiety	0.010	0.017	0.016	0.606	0.544	−0.063				
	Stress	−0.001	0.018	−0.001	−0.048	0.962	−0.082				
	SLSQ-L	0.117	0.012	0.168	9.937	<0.001	0.185				
Model 4	(Constant)	9.932	2.556		3.886	<0.001		3.11	0.027	23.31	<0.001
	Sleep latency	0.003	0.006	0.030	0.498	0.618	−0.102				
	Number of hours of sleep	−0.024	0.044	−0.013	−0.536	0.592	0.043				
	Sleep efficiency	0.038	0.028	0.086	1.353	0.176	0.114				
	Sleep quality	0.355	0.076	0.089	4.646	<0.001	0.144				
	ISI	−0.037	0.011	−0.068	−3.475	0.001	−0.139				
Model 5	(Constant)	11.383	0.265		42.987	<0.001		3.03	0.073	79.58	<0.001
	Sitting time	−0.068	0.016	−0.066	−4.333	<0.001	−0.080				
	IPAQ score	<0.001	<0.001	0.114	7.410	<0.001	0.143				
	SSPQ-L	0.068	0.005	0.219	14.237	<0.001	0.237				
	Technology use behavior	0.014	0.018	0.012	0.800	0.424	0.010				
Model 6	(Constant)	13.706	0.328		41.738	<0.001	13.706	3.02	0.084	27.23	<0.001
	MedDiet awareness	1.569	0.098	0.248	16.043	<0.001	0.260				
	Barriers: Food allergies and intolerances	−0.170	0.142	−0.018	−1.197	0.231	−0.003				
	Barriers: Cultural and/or religious limitations	−0.162	0.162	−0.016	−1.000	0.318	0.003				
	Barriers: Medical/health-related limitations	−0.256	0.161	−0.025	−1.590	0.112	−0.044				
	Barriers: Individual beliefs limitation (e.g., vegan and vegetarian)	−0.680	0.170	−0.062	−4.010	<0.001	−0.066				
	Barriers: Taste dislike	−0.544	0.126	−0.067	−4.314	<0.001	−0.087				
	Barriers: Attitudes (suitability, taste, restrictive, food waste)	−0.133	0.128	−0.016	−1.040	0.298	0.003				
	Barriers: Social norms (food culture)	−0.277	0.132	−0.033	−2.104	0.035	−0.026				
	Barriers: Low motivation	−0.487	0.131	−0.059	−3.718	<0.001	−0.087				
	Barriers: Price unaffordability	−0.230	0.111	−0.033	−2.071	0.038	−0.013				
	Barriers: Time/effort-consuming	−0.361	0.116	−0.049	−3.109	0.002	−0.054				
	Barriers: Low accessibility/availability of Mediterranean food	−0.319	0.146	−0.035	−2.183	0.029	−0.019				
	Barriers: Lack of knowledge and cooking skills	−0.231	0.130	−0.028	−1.785	0.074	−0.061				
	Barriers: Others	−0.407	0.328	−0.019	−1.239	0.215	−0.017				

UCs: unstandardized coefficients; SCs: standardized coefficients; SEE: standard error of the estimate; R: coefficient of correlation. R^2^: adjusted coefficient of determination; SLSQ-L: Short Life Satisfaction Questionnaire; ISI: Insomnia Severity Index; IPAQs: International Physical Activity Questionnaires; SSPQ-L: Short Social Participation Questionnaire.

**Table 3:** Comprehensive predictive models of adherence to MedLifestyle.

		UC		SC	T	Sig.	R	SEE	Adj. R^2^	ANOVA	
		B	SE	β						F	*p*-Value
Model 7	(Constant)	8.047	0.585		13.765	<0.001		2.85	0.179	44.872	<0.001
	Age	0.016	0.003	0.077	4.545	<0.001	0.061				
	Health	0.148	0.081	0.028	1.818	0.069	0.066				
	Education	0.11	0.036	0.046	3.064	0.002	0.09				
	Living environment	−0.227	0.062	−0.054	−3.676	<0.001	−0.066				
	Region	0.352	0.099	0.053	3.564	<0.001	0.083				
	Continent	0.009	0.075	0.002	0.114	0.909	−0.045				
	SLSQ-L	0.06	0.011	0.086	5.494	<0.001	0.187				
	Sleep quality	0.291	0.071	0.073	4.085	<0.001	0.144				
	ISI	−0.014	0.01	−0.026	−1.404	0.16	−0.139				
	SSPQ-L	0.061	0.005	0.196	12.299	<0.001	0.237				
	Sitting time	−0.057	0.015	−0.055	−3.806	<0.001	−0.08				
	IPAQ score	<0.001	<0.001	0.095	6.46	<0.001	0.143				
	MedDiet awareness	1.297	0.101	0.205	12.901	<0.001	0.26				
	Barriers: Individual beliefs limitation (e.g., vegan and vegetarian)	−0.534	0.161	−0.049	−3.315	0.001	−0.066				
	Barriers: Taste dislike	−0.528	0.12	−0.065	−4.411	<0.001	−0.087				
	Barriers: Social norms (food culture)	−0.144	0.125	−0.017	−1.157	0.247	−0.026				
	Barriers: Low motivation	−0.372	0.123	−0.045	−3.01	0.003	−0.087				
	Barriers: Price unaffordability	−0.392	0.105	−0.056	−3.73	<0.001	−0.013				
	Barriers: Time/effort-consuming	−0.341	0.11	−0.046	−3.102	0.002	−0.054				
	Barriers: Low accessibility/availability of Mediterranean food	−0.337	0.138	−0.037	−2.441	0.015	−0.019				
Model 8	(Constant)	7.93	0.43		18.453	<0.001		2.85	0.179	55.55	<0.001
	Age	0.014	0.003	0.071	4.446	<0.001	0.061				
	Education	0.118	0.036	0.049	3.299	0.001	0.09				
	Living environment	−0.232	0.062	−0.055	−3.763	<0.001	−0.066				
	Region	0.382	0.096	0.058	3.958	<0.001	0.083				
	SLSQ-L	0.065	0.011	0.093	6.032	<0.001	0.187				
	Sleep quality	0.359	0.06	0.089	5.978	<0.001	0.144				
	SSPQ-L	0.061	0.005	0.194	12.36	<0.001	0.237				
	Sitting time	−0.059	0.015	−0.057	−3.931	<0.001	−0.08				
	IPAQ score	<0.001	<0.001	0.096	6.576	<0.001	0.143				
	MedDiet awareness	1.3	0.096	0.206	13.523	<0.001	0.26				
	Barriers: Individual beliefs limitation (e.g., vegan and vegetarian)	−0.523	0.16	−0.048	−3.258	0.001	−0.066				
	Barriers: Taste dislike	−0.547	0.119	−0.067	−4.595	<0.001	−0.087				
	Barriers: Low motivation	−0.377	0.123	−0.045	−3.075	0.002	−0.087				
	Barriers: Price unaffordability	−0.387	0.105	−0.055	−3.694	<0.001	−0.013				
	Barriers: Time/effort-consuming	−0.345	0.11	−0.047	−3.143	0.002	−0.054				
	Barriers: Low accessibility/availability of Mediterranean food	−0.333	0.136	−0.036	−2.439	0.015	−0.019				

UCs: unstandardized coefficients; SCs: standardized coefficients; SEE: standard error of the estimate; R: coefficient of correlation. R^2^: adjusted coefficient of determination; SLSQ-L: Short Life Satisfaction Questionnaire; ISI: Insomnia Severity Index; IPAQs: International Physical Activity Questionnaires; SSPQ-L: Short Social Participation Questionnaire.

## Data Availability

The datasets generated and analyzed during the current study are not publicly available at this time as further analyses are ongoing, and additional publications based on these data are in preparation. Data may be made available upon reasonable request to the corresponding author once all planned analyses and publications are completed.
